# Body Water-Mediated Conductivity Actualizes the Insect-Control Functions of Electric Fields in Houseflies

**DOI:** 10.3390/insects11090561

**Published:** 2020-08-23

**Authors:** Yoshihiro Takikawa, Takeshi Takami, Koji Kakutani

**Affiliations:** 1 Plant Center, Institute of Advanced Technology, Kindai University, Wakayama 642-0017, Japan; takikawa@waka.kindai.ac.jp; 2 Department of Internal Medicine, Clinic Jingumae, Nara 634-0804, Japan; takami66@m5.kcn.ne.jp; 3 Pharmaceutical Research and Technology Institute and Anti-Aging Centers, Kindai University, Osaka 577-8502, Japan

**Keywords:** attractive force, discharge exposure, insect trapping, insect dismemberment, housefly, static electric field, dynamic electric field, discharged-mediated positive electrification

## Abstract

**Simple Summary:**

The attractive forces generated in a static electric field, as well as corona and arc discharges generated in a dynamic electric field, are practical approaches for trapping and killing insects that enter the electric field. These electrostatic methods are realized by the conductive nature of the insect body. Thus, this work examined the role of body water on the conduction of electricity in the insect body. Adult houseflies (*Musca domestica*) were subjected to dehydration, rehydration, refrigeration, and freezing and thawing. These insects were then placed in static and dynamic electric fields to examine whether the release of negative charges from the insect caused attraction in the static field, as well as whether the fly was heated or dismembered when electricity passed through its body in a dynamic field. There was no current in the bodies of dehydrated and frozen flies; hence, there was no attractive force or discharge exposure. In the remaining insects, the results were identical to those in the control insects. Therefore, the conduction of electricity in the insect’s body water enables the insect-control effects of the electric fields.

**Abstract:**

In the present study, the relationship between body water loss and conductivity was examined in adult houseflies (*Musca domestica*). The events an insect experiences in an electric field are caused by the conductive nature of the insect body (i.e., movement of electricity within or its release from the insect). After houseflies were dehydrated, rehydrated, refrigerated, and frozen and thawed, they were placed in static and dynamic electric fields. Untreated houseflies were deprived of their free electrons to become positively charged and then attracted to the insulated negative pole in the static electric field and were exposed to corona and arc discharge from non-insulated negative pole in the dynamic electric field. There was no current in the bodies of dehydrated and frozen flies; hence, there was no attractive force or discharge exposure. In the remaining insects, the results were identical to those in the untreated control insects. These results indicated that the reduction of body water conductivity inhibited the release of electricity from the body in the static electric field and the discharge-mediated current flow through the body in the dynamic electric field. The insect was affected by the electric fields because of its conductivity mediated by body water.

## 1. Introduction

Electrostatic phenomena that occur in electric fields have been used to manage insects, pathogens, and weeds as an alternative to pesticides, due to pesticide resistance and public demand to reduce overall pesticide use [1]. Electrostatic engineering has been applied to the design, construction, and control of electrical equipment that generates different electric fields, defined as the space surrounding an electric charge within which it is capable of exerting a perceptible force on another electric charge [2]. There are many ways to generate electric fields. Typically, a negatively or positively charged conductor is placed at a distance from a grounded conductor to create a potential difference [3]. With larger voltages, the electric field becomes more powerful because of the greater potential difference. When an insulated conductor is used, a non-discharging electric field (i.e., no electric current between the two conductors) is produced [3]. Conversely, a non-insulated charged conductor produces a discharge-generating electric field, in which corona and arc discharges occur depending on the distance and potential difference between the two conductors [3].

A non-discharging electric field can generate a strong attractive force in an electrostatic field [4,5,6] or a static electric field [7,8]. These attractive forces can function as an electric field screen that harnesses the nature of an electric field [3]. Apparatuses constructed based on this principal can be used to prevent the entry of airborne spores of pathogens [4,5,6], flying insects [7,8], pollen grains that cause pollenosis [9], and fine smoke particles [10]. In addition to capturing insects, an electric field screen will repel many insects that inherently avoid entry into a static electric field [8,9,10,11,12]. Electric fields that generate a discharge can be used to kill insects that enter the field by direct, instantaneous exposure to an arc discharge [13,14].

In a static electric field, a negative charge will strongly repulse other negative charges (i.e., free electrons), pushing them out of the insect body toward the ground. This causes the insect to become positively charged, attracting it to the negative charge of the conductor [15,16,17,18]. Insects that enter a discharge-generating electric field are selectively subjected to arc discharge from the non-insulated charged conductor, due to its higher between-conductor conductivity [14]. The ability of an insect to avoid a static electric field depends on its ability to perceive the electric field. Newland et al. [19] reported that cockroaches could detect an electrostatic field with their antennae. When subjected to an electric field, cockroaches deflected their antennae against the attraction forces, moving their antennae toward the electrode. Nonomura et al. [11] reported that whiteflies placed their antennae inside the electric field formed between opposite poles. This was similar to “searching” behavior and the insects were deterred from entering the electric field. Antennae appear to be influenced by the repulsive electric force, leading to an uneven distribution of electricity in the organ as a result of electrostatic induction [19].

The events an insect experiences in an electric field are caused by the conductive nature of the insect body (i.e., movement of electricity within or its release from the insect). However, it is unclear how electricity is conducted within the insect body. Many studies have reported that the cuticle (the outer protective layer that covers the bodies of many invertebrates) is efficiently charged because of its highly conductive nature [20,21,22,23,24]. We inferred that the insects used in our previous studies preferentially underwent various electrostatic events, such as attraction and discharge exposure, due to the high conductivity of their cuticles [15,16,17,18]. However, we frequently observed that some dead insects were not attracted or not subject to discharge exposure when they were placed in an electric field. This led us to reconsider insect body conductivity. If our previous interpretation had been correct, the cuticle layer should have remained conductive after an insect died.

Therefore, this study examined the relationship between body water loss and conductivity; a preliminary experiment showed that dying insects were likely to become less attractive in a static electric field and less-exposed in a discharge-generating electric field. We constructed instruments to produce static and dynamic electric fields. Using insects that had lost different proportions of body water or that had been frozen, we examined changes in body conductivity in both electric fields. Based on the results, we discuss the possibility that body water conducts electricity in the insect body.

## 2. Materials and Methods

### 2.1. Test Insect

Adult (body length 8–10 mm) houseflies (*Musca domestica*) were studied. Housefly pupae were purchased from Sumika Technoservice (Hyogo, Japan) and maintained in a growth chamber (25.0 ± 0.5 °C, 12-h photoperiod at 4000 lux) using our standard method. Newly emerged adult houseflies with the same body lengths were used in all experiments. The average weight of 30 adult houseflies was 38.2 ± 2.3 mg.

### 2.2. Temperature Treatments of Insects

Four groups of experimental insects were prepared: (1) insects dehydrated to different degrees, (2) insects rehydrated after desiccation, (3) insects kept in cold storage, and (4) insects frozen and then thawed. Untreated insects were used as a control.

The first group of insects was prepared using the loss-on-drying (LOD) method [25]. Insects were weighed and placed in a thermostat convection oven set at 30 °C for dehydration. At intervals, insects were removed from the oven and weighed. This procedure was continued until the weight remained constant. Then, the difference between the initial and final weights was calculated to determine the moisture (body water) vaporized. Using a weight-loss calibration curve, insects that lost different proportions of body water were collected and used for the experiment. Insects that had lost 30%, 50%, and 80% of their body water were designated as D_30_-, D_50_-, and D_80_-insects, respectively.

The rehydrated insects in the second group were prepared by infiltration of a specific volume of water into a dried insect to restore its original body weight. The insects were immersed in a definite volume of water until the water completely infiltrated the body. When D_80_-insects were used, they were designated D_80_M-insects.

The third group of insects was prepared by placement in a refrigerator at 4 °C for 2 h to induce a state of temporary dormancy. These refrigerated insects were designated R-insects. R-insects were used in the experiments before they awoke.

The insects in the fourth group were placed in a freezer (−20 °C) for 2 h to achieve a frozen state, then used in the experiments. These insects were designated F-insects. Some F-insects were placed at room temperature for 1 h to thaw before they were used in experiments. These insects were designated FT-insects.

The insects that were sprayed with quick-acting pyrethroid (Earth jet, Earth Corporation, Tokyo, Japan) and neonicotinoid (Bestguard, Sumitomo Chemical Garden Products, Tokyo, Japan) insecticides were used for comparative experiment. These chemically killed insects were designated as CKp- and CKn-insects, respectively. These insects were used in experiments immediately after they died.

### 2.3. Construction of the Static (SEP) and Dynamic (DEP) Electric Field Producers

**Figure 1:**
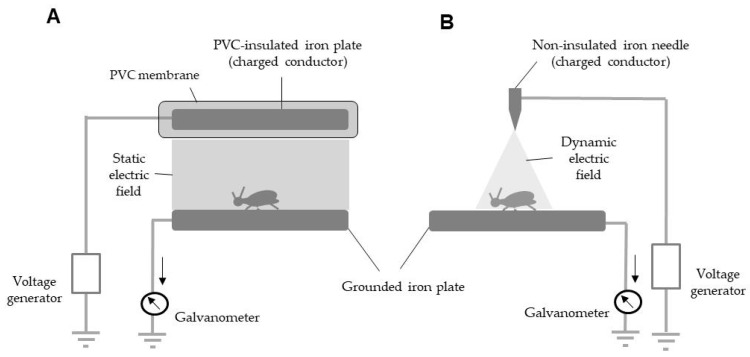
Schematic representations of static (**A**) and dynamic (**B**) electric field producers. Using an insect aspirator, a test insect was transferred onto a grounded plate in the electric field. Arrow indicates direction of electricity movement.

Figure 1 shows the SEP and DEP. The SEP consisted of two identical iron plates (20 × 200 mm^2^) arranged in parallel, 9 mm apart; one was insulated with a polyvinylchloride membrane (1 mm thick, 10^9^ Ωcm), linked to a direct-current voltage generator (Max Electronics, Tokyo, Japan) and negatively charged with different voltages (Figure 1A). The DEP consisted of a non-insulated pointed iron rod (5 mm diameter, 100 mm long) linked to the same voltage generator and a grounded iron plate (Figure 1B). In both situations, a galvanometer (Sanwa, Tokyo, Japan) was integrated in the ground line of the grounded plate to measure the current to the ground via the grounded plate. The electric current profiles were recorded with a current detector (detection limit 0.01 µA) integrated into the galvanometer.

### 2.4. Measurement of Attraction of Dehydrated Insects in the SEP and Electric Current Released from the Insects

In the first experiment, the voltage applied to the insulated iron plate of the SEP was raised gradually to determine the highest voltage that caused a mechanical discharge from the charged plate (i.e., current from the charged insulated conductor to the grounded plate through silent discharge) in the absence of a test insect. In subsequent experiments, insect attraction and electric current generation were examined using voltages (−5.0 to −7.5 kV) that caused no mechanical discharge.

In the second experiment, the insulated plate was negatively charged with different voltages; D-, DM-, R-, F-, and FT-insects were transferred to the grounded plate to examine whether they were attracted to the charged plate. Insect attraction to the charged plate was video-recorded. Simultaneously, the transient electric current from the insect was recorded with the current detector in the galvanometer. Untreated insects were used as a positive control.

### 2.5. Insect Exposure to Corona and Arc Discharges

Dehydrated and untreated insects were placed on the grounded plate of the DEP; the voltage applied to the needle conductor was then gradually increased to determine the lowest voltage that resulted in corona or arc discharge between the needle tip and insect body. The continuous electric current to the ground through the corona discharge was recorded in a manner similar to the method mentioned above. The insects were weighed before and after corona discharge to determine the loss of body water. Insects exposed to corona and arc discharges were video recorded.

## 3. Results and Discussion

Electric fields create various fascinating phenomena that can be used to control insect pests. Consequently, considerable effort has expended to understand the principles of target phenomena and develop instruments to generate electric fields [26]. The attractive force generated in a static electric field and the discharge generated in a dynamic electric field have been adopted as practical strategies for pest control [1,3,26]. These physical measures can capture or dismember insects that enter the electric fields [7,8,14]. Theoretically, the attractive force is generated as a result of positive electrification of the insect body due to the loss of electrons from the body [15,16,17,18]. The corona and arc discharge exposure then conducts electricity through the insect body [13,14]. These two phenomena depend on the conductive nature of the insect body. Therefore, this work examined the entity responsible for this conductive nature, focusing on the body water of adult houseflies.

### 3.1. Assessment of Body Water Loss Using LOD Desiccation

**Figure 2:**
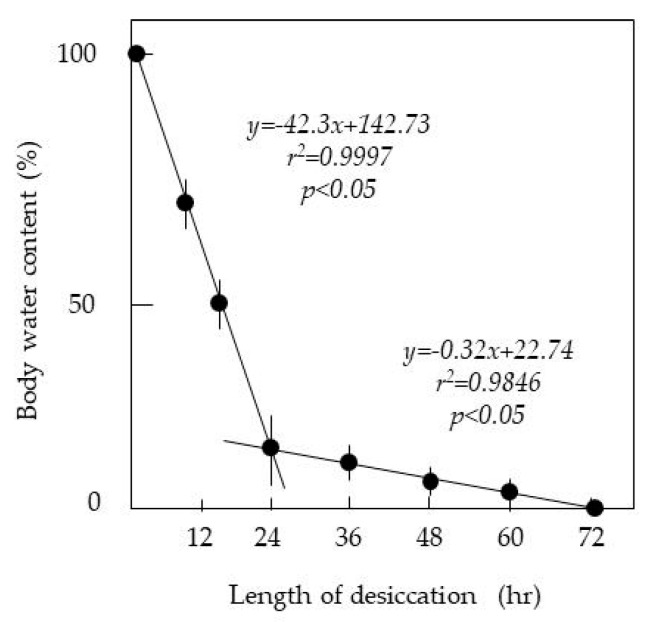
Relationship between the duration of desiccation and loss of body water in adult houseflies with the LOD method. Twenty insects were used for each desiccation duration.

The desiccation resistance of insects is measured by the change in mass (body weight) on drying; the mass difference between before and after drying is attributed to body water loss [27]. Figure 2 shows the temporal change in the weights of test insects. The technique used in this study effectively dehydrated the insects to the desired levels by changing the duration of desiccation. The data exhibited a high degree of reproducibility. There were two distinct phases in the loss of body water: an initial rapid loss, followed by a slower second phase. In both phases, there was a linear relationship between the length of desiccation and the extent of body water loss in the insects. The total body water of an adult housefly constituted 40–45% of the total body weight. Water may also become locked in molecular structures as bound moisture; greater amounts of heat energy are needed to release the tightly bound moisture. Our LOD treatment likely promoted the vaporization of free water in the insect body or at the body surface.

### 3.2. Experimental Instrument Reflecting Electrostatic Principles of Electric Field Screens

Two electric field screens were constructed to control insect pests [1,3]. Both screens had a similar grounded metal net and differed in the configuration of the charged conductors: one configuration comprised a layer of insulated conductor wires arrayed in parallel to form a static electric field against the grounded metal net [8,11], while the other configuration comprised a non-insulated perforated metal plate with multiple metal needles to form a dynamic electric field against the grounded net [13]. Using the static electric field [11], pests that reached the net stopped and extended their antennae or legs into the static electric field, similar to “searching” behavior. These insects did not enter the static electric field. However, when insects were blown onto the net, most assumed postures to avoid the wind; some could not maintain balance and were forcibly pushed inside the screen. These insects were captured by the strong force of the electric field. In the dynamic electric field screen [13], an insect reaching the grounded net entered the electric field without hesitation. It was immediately subjected to an arc discharge without experiencing attraction to the charged non-insulated pole. The structures of our SEP and DEP reflect the main characteristics of these electric field screens [3].

### 3.3. Mechanical Discharge of the SEP

**Figure 3:**
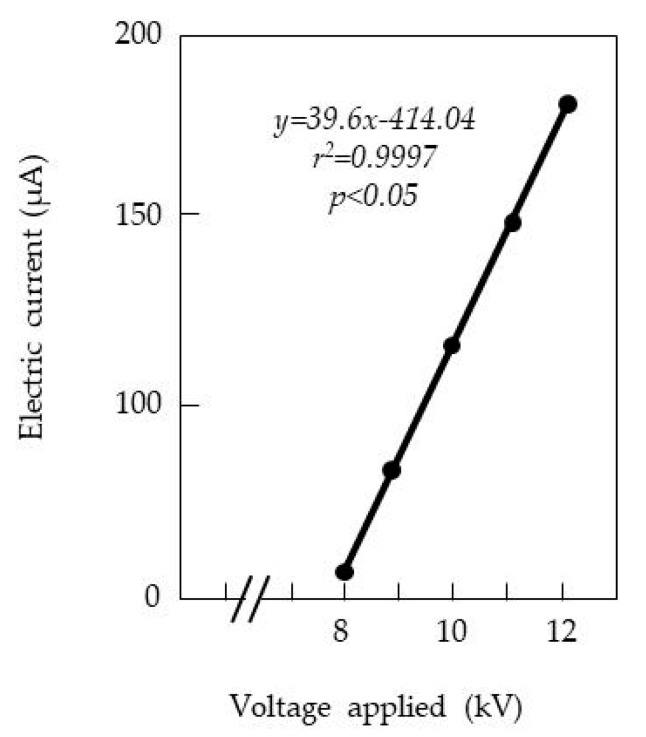
Relationship between voltage applied to the insulated plate and current magnitude produced through corona discharge in the electric field of the SEP.

The SEP produced a static electric field between the charged insulated plate and grounded non-insulated plate (Figure 1A). In this field, the discharge (i.e., electric current between the plates) was intercepted by the material insulating the charged conductor and the air between two plates; the resulting electrical resistance impeded the movement of electricity on the surface of the charged plate to a ground (via the grounded plate). However, excess voltage overcame this resistance, causing corona and arc discharges in the electric field [28]. Corona discharges were induced first, followed by arc discharges at higher applied voltages. To block these discharges, it was essential to determine the lowest voltage causing a corona discharge (silent discharge) in our instrument (Figure 3).

Within the voltage range used in our study, a discharge was first induced when −8.0 kV was applied to the insulated plate; the electric current increased in accordance with the applied voltage. Based on these results, the insect attraction assay with the SEP was conducted using voltages below −7.6 kV.

### 3.4. Attraction of Treated Insects in the SEP and Detection of Their Transient Electric Current Discharges

**Figure 4:**
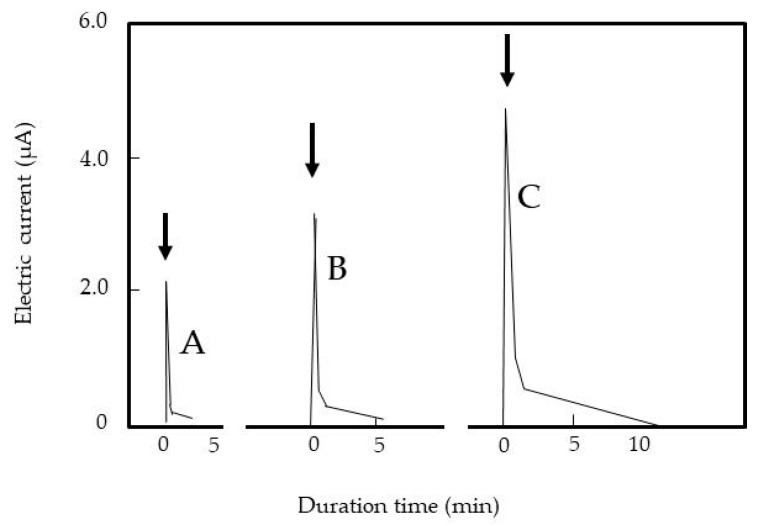
Transient electric currents from discharges of adult houseflies when the insulated plate of the SEP has a charge of (A) −6.5, (B) −7.0, and (C) −7.5 kV. Arrow indicates when insect was placed on grounded plate.

Supplementary Video S1 shows the attraction of a housefly to a negatively charged insulated plate. When the insect was placed on the grounded plate, it was rapidly attracted to the charged plate. The force of the charged plate that captured the insect was strong, such that the insect could not escape. Figure 4 shows the profile of the transient electric current generated at the time of attraction. A sharp peak in the transient electric current was detected in the test insects, and a greater electric current was produced when larger voltages were applied. In the following experiment, temperature-treated insects were used for comparative analysis.

Table 1 lists the proportions of treated and untreated insects attracted at different voltages, as well as the greatest magnitude of the transient electric current at the time of attraction. Based on the results for untreated insects (N-insect, positive control), we concluded that larger voltages forced more negative electrons out of the insect; this caused the insects to exhibit a greater positive charge and be captured more strongly by the oppositely charged insulated plate. This finding supports the insect attraction hypothesis (discharge-mediated positive electrification) [15,16,17,18]. A loss of body water through desiccation reduced current flow from the insect (i.e., in D_30_-, D_50_-, and D_80_-insects). The current flow stopped when 30% of body water was lost (i.e., in D_30_-insects), strongly suggesting that body water conducts electricity in insects. At this level of dehydration, the insects were dead. However, the conductivity of the dried insect was recovered by infiltrating the body with water (i.e., in DM-insects). Moreover, the current occurred normally. Therefore, the conductive nature of the insect was independent of its survival or active/dormant state. Notably, the current completely disappeared when the body was frozen; it recovered when the frozen body was thawed. Electrical conduction requires the flow of charged particles. In water, these charge carriers are ions from dissolved salts or disassociated water molecules. When water freezes, the ions are much less able to move freely (i.e., ice becomes insulative) [29,30,31]. Thus, the change in conductivity between the frozen and thawed insects reflects the physical nature of water and ice. In this experiment, we used the insects killed by quick-acting neurotoxic insecticides for comparison (Table 1). These dead insects (not dehydrated) were shown to be highly conductive, such that they were attracted to the negatively charged insulated plate. The data were very close to those of N- and R-insects. These results strongly support the present conclusion mentioned above. Nevertheless, the present results may be insufficient to completely deny the involvement of cuticle in the electric conduction in the insect body. In fact, the conductivity in D_80_M- and FT-insects was significantly lower than that of N-insect. This nature was conspicuous in the application of lower voltages (6 and 6.5 kV). Obviously, both insects remained less-conductive even after they were rehydrated to their original levels. These results suggest that another factor supplementary to body water conductivity could be involved. A possible interpretation was the irreversible disintegration of electrically conductive cuticular structures by drastic treatments of freezing and dehydration. This problem is the major target in out next study.

### 3.5. Exposure of Treated Insects to Corona and Arc Discharges in the DEP

Videos S2 and S3 show insects exposed to corona and arc discharges from the charged needle conductor, respectively. Corona discharge exposure continued until the insect body became dried due to heating. In contrast, insects exposed to arc discharges were dismembered by the strong impact of the arc discharge.

Table 2 shows the initial magnitude of the electric current through insects exposed to corona discharge. Corona discharges were detected in N-, D_30_-, D_50_-, D_80_M-, R-, and FT-insects, but not in D_80_- or F-insects. As the applied voltage increased, the magnitude of the electric current increased with larger corona discharges. The body water loss of exposed insects was similar in all dehydrated insects.

Table 3 shows the proportions of treated insects that were exposed to arc discharges at different voltages. Larger voltages had a greater effect on the insect and more insects were exposed to these discharges. The electric current of arc discharges was not recorded because the current immediately exceeded the detection limit of the current detector.

In summary, the discharge of the charged conductor in a dynamic electric field was the movement of charge accumulated on the conductor to the ground through the insect body. Namely, an electric current was generated between opposite poles due to dielectric breakdown in the electrical field related to the potential difference between the poles [28]. This current was affected by the reduced conductivity of the insect body (i.e., enhanced body resistivity), due to partial loss or freezing of body water. Using this interpretation, the loss of insect attraction in the static electric field could be explained as follows: the reduction of body water conductivity inhibited the release of electricity from the body, which caused the insect body to lose force (positive electrification) moving toward the oppositely charged insulated conductor. The insect was affected by the electric fields because of its conductivity mediated by body water.

In our previous paper, we demonstrated that in a static electric field, different kinds of insects were similarly attracted to a negatively charged conductor as a result of positive electrification of the insect body due to the loss of electrons from the body [17]. The insects tested were tomato leaf-minor fly (*Liriomyza sativae*), greenhouse shore fly (*Scatella stagnalis*), bath room fly (*Clogmia albipunctatus*), Asian tiger mosquito (*Aedes albopictus*) (Diptera); green peach aphid (*Myzzus percicae*), whitefly (*Bemisia tabaci*), green rice leafhopper (*Nephotettix cincticeps*) (Hemiptera); rice weevil (*Sitophilus oryzae*), red flour beetle (*Tribolium castaneum*), adzuki bean weevil (*Callosobruchus chinensis*) (Coleoptera); oriental termite (*Coptotermes formosanas*) (Isoptera); book louse (*Liposcelis bostrychophilus*) (Psocoptera); German cockroach (*Blattella germanica*) (Blattodea); common clothes moth (*Tineola bisselliella*) (Lepidoptera); western flower thrips (*Franklinella occidentalis*) (Thysanoptera). All of these insects were subjected to electric discharge when they entered a discharge-generating electric field. More importantly, these insects lost body conductivity when they were dehydrated or frozen (unpublished data). These results strongly suggest that the conclusion obtained in this work is applicable to a wide range of insect species.

## 4. Conclusions

Insects were subject to dehydration and freezing to reduce their body water conductivity. Neither dried nor frozen insects developed an attractive force or discharge exposure in the static and dynamic electric fields. Importantly, the rehydrated and thawed insects showed results identical to those of untreated normal insects, suggesting that insect conductivity depends on the physical nature of water, regardless of insect survival. Therefore, body water contributes to the conductive nature of insects, which enables insect control by electric fields.

## Figures and Tables

**Table 1:** Percentages of treated and untreated houseflies attracted to the charged insulated plate in a static electric field (SEP) and greatest magnitude of the insect-derived transient current generated at the time of attraction.

**Types of Insects ^a^**	**Percentage of Attracted Insects**					
	**5**	**5.5**	**6**	**6.5**	**7**	**7.5 (kV)**
N-insect	0	18.0 ± 2.7 ^V^	64.0 ± 8.2 ^V^	99.0 ± 2.2 ^V^	100 ^V^	100 ^V^
D_30_-insect	0	0	0	0	0	75.0 ± 7.9 ^W^
D_50_-insect	0	0	0	0	0	0
D_80_-insect	0	0	0	0	0	0
D_80_M-insect	0	0	0	61.0 ± 8.2 ^W^	93.0 ± 5.7 ^W^	100 ^V^
R-insect	0	16.0 ± 5.5 ^V^	58.0 ± 2.7 ^V^	98.0 ± 4.5 ^V^	100^V^	100 ^V^
F-insect	0	0	0	0	0	0
FT-insect	0	0	13.0 ± 8.4 ^W^	71.0 ± 8.2 ^W^	100 ^V^	100 ^V^
CKp-insect	0	17.6 ± 2.5 ^V^	63.0 ± 9.7 ^V^	98.0 ± 2.7 ^V^	100 ^V^	100 ^V^
CKn-insect	0	17.4 ± 2.4 ^V^	62.6 ± 9.6 ^V^	96.4 ± 3.3 ^V^	100 ^V^	100 ^V^
**Types of Insects ^a^**	**Greatest Magnitude (µA) of Electric Current**					
	**5**	**5.5**	**6**	**6.5**	**7**	**7.5 (kV)**
N-insect	0.8 ± 0.1 ^V^	1.1 ± 0.2 ^V^	1.6 ± 0.2 ^V^	2.1 ± 0.2 ^V^	2.6 ± 0.3 ^V^	4.5 ± 0.6 ^V^
D_30_-insect	0	0	0.2 ± 0.1 ^W^	0.4 ± 0.2 ^W^	0.9 ± 0.2 ^W^	1.8 ± 0.4 ^W^
D_50_-insect	0	0	0	0.1 ± 0.1 ^W^	0.3 ± 0.1 ^X^	0.5 ± 0.1 ^X^
D_80_-insect	0	0	0	0	0	0
D_80_M-insect	0.1 ± 0.1 ^W^	0.3 ± 0.1 ^W^	0.7 ± 0.1 ^X^	1.6 ± 0.3 ^X^	2.3 ± 0.3 ^V^	3.9 ± 0.4 ^V^
R-insect	0.7 ± 0.2 ^V^	1.1 ± 0.3 ^V^	1.5 ± 0.2 ^V^	2.1 ± 0.3 ^V^	2.5 ± 0.3 ^V^	4.4 ± 0.4 ^V^
F-insect	0	0	0	0	0	0
FT-insect	0.2 ± 0.1 ^W^	0.3 ± 0.2 ^W^	1.1 ± 0.4 ^X^	1.5 ± 0.7 ^X^	2.4 ± 0.6 ^V^	3.3 ± 0.9 ^V^
CKp-insect	0.8 ± 0.3 ^V^	1.1 ± 0.4 ^V^	1.6 ± 0.1 ^V^	2.1 ± 0.5 ^V^	2.6 ± 0.6 ^V^	4.5 ± 0.8 ^V^
CKn-insect	0.8 ± 0.4 ^V^	1.0 ± 0.5 ^V^	1.6 ± 0.3 ^V^	2.1 ± 0.2 ^V^	2.6 ± 0.3 ^V^	4.5 ± 0.6 ^V^

^a^ Test insects were classified by dehydration treatments: N-insect, untreated insect (positive control); D_30-80_-insects, dehydrated insects that had lost 30–80% of their body water; D_x_M-insect, a dried and rehydrated insect; R-insect, insect kept in a refrigerator at 4 °C; F-insect, insect frozen at −20 °C; FT-insect, an insect frozen and then thawed. CKp- and CKn-insect, an insect killed with pyrethroid and neonicotinoid insecticide, respectively. Twenty insects were used for each condition; the means and standard deviations were calculated from five replicates of each experiment. Letters V–X with means in each vertical column indicate significant differences (*p* < 0.05) according to Tukey’s method.

**Table 2:** Initial magnitude of electric current through temperature-treated houseflies exposed to a corona discharge in the dynamic electric field producer (DEP) negatively charged with different voltages.

Types of Insects ^a^	Initial Magnitude (µA) of Electric Current				
	8	8.5	9	9.5	10 (kV)
N-insect	0	10.7 ± 1.9 ^V^	125.2 ± 21.0 ^V^	176.4 ± 16.9 ^V^	257.4 ± 27.1 ^V^
D_30_-insect	0	0	0	8.6 ± 2.5 ^W^	80.5 ± 14.6 ^W^
D_50_-insect	0	0	0	0	8.9 ± 2.4 ^X^
D_80_-insect	0	0	0	0	0
D_80_M-insect	0	7.8 ± 2.4 ^V^	93.4 ± 12.8 ^V^	143.1 ± 17.6 ^V^	220.4 ± 32.0 ^V^
R-insect	0	9.3 ± 2.1 ^V^	116.5 ± 17.0 ^V^	165.8 ± 13.8 ^V^	250.4 ± 39.0 ^V^
F-insect	0	0	0	0	0
FM-insect	0	8.6 ± 1.6 ^V^	105.9 ± 19.3 ^V^	154.5 ± 22.2 ^V^	241.8 ± 33.1 ^V^

^a^ See legend of Table 1. Twenty insects were used for each condition; the means and standard deviations were calculated from five replicates of each experiment. Letters V–X with means in each vertical column indicate significant differences (*p* < 0.05) according to Tukey’s method.

**Table 3:** Percentage of temperature-treated houseflies exposed to an arc discharge in the dynamic electric field producer (DEP).

Types of Insects ^a^	Voltage (KV) Applied to the Non-insulated Pointed Conductor			
	10.5	11	11.5	12
N-insect	97.0 ± 2.7 ^V^	100	100 ^V^	100 ^V^
D_30_-insect	0	0	18.0 ± 8.4 ^W^	46.0 ± 7.4 ^W^
D_50_-insect	0	0	0	18.0 ± 6.7 ^X^
D_80_-insect	0	0	0	0
D_80_M-insect	82.0 ± 6.7 ^W^	100	100 ^V^	100 ^V^
R-insect	93.0 ± 2.7 ^W^	100	100 ^V^	100 ^V^
F-insect	0	0	0	0
FM-insect	88.0 ± 6.7 ^W^	100	100 ^V^	100 ^V^

^a^ See legend of Table 1. Twenty insects were used for each condition; the means and standard deviations were calculated from five replicates of each experiment. Letters V-X with means in each vertical column indicate significant differences (*p* < 0.05) according to Tukey’s method.

## Supplementary Materials

The following are available online at https://www.mdpi.com/2075-4450/11/9/561/s1, Video S1: Adult housefly attracted to the charged insulator plate of the static electric field producer (−7.5 kV charge), Video S2: Adult housefly exposed to a corona discharge from a negatively charged needle probe (non-insulated) of the dynamic electric field producer (−10 kV charge), Video S3: Adult housefly exposed to an arc discharge from a negatively charged needle probe (non-insulated) of the dynamic electric field producer (−12 kV charge).
